# Distal femoral stem-bone anchorage of a cementless revision total hip arthroplasty: Evaluation of 14 patients by CT

**DOI:** 10.3109/17453670903039403

**Published:** 2009-06-01

**Authors:** Rüdiger J Weiss, Fredrik Strömwall, Mats O Beckman, Karl A Hansson, André Stark

**Affiliations:** ^1^Section of Orthopaedics and Sports Medicine, StockholmSweden; ^2^Section of Radiology, Department of Molecular Medicine and Surgery, Karolinska InstitutetStockholmSweden

## Abstract

**Background and purpose** According to the manual of the cementless Link MP reconstruction prosthesis, a distal femoral stem-bone anchorage of at least 80 mm is necessary to gain implant stability. There have been no in vivo studies showing that this distance is either achieved in clinical practice or needed for clinically satisfying results. Thus, we assessed the femoral stem-bone anchorage of the MP prosthesis using CT.

**Methods** 14 patients with the MP stem were evaluated by CT scans at a median follow-up time of 12 months postoperatively. Femoral stem-bone anchorage was defined as adequate if 50% of the stem flutes or more had cortical bone contact. The length of anchorage was derived from the number of slices with adequate anchorage. Clinical outcome was assessed with VAS for pain and Harris hip score (HHS), both at 1 and 5 years of follow-up.

**Results** The median length of stem-bone anchorage was 33 mm (interquartile range 10–60), which was shorter than recommended (p = 0.002). Still, at the 1-year control, all patients were fully weight-bearing and only 1/14 complained about mild thigh pain. 7/14 patients did not experience any pain in the affected hip. The patients had a median of 85 points in the HHS. The clinical outcome at 5 years was unchanged.

**Interpretation** We found that it can be difficult to achieve a stem-bone anchorage of at least 80 mm for the MP Link prosthesis. However, this does not appear to be necessary to obtain stability and to achieve clinically satisfying results.

## Introduction

Fluted, tapered cementless prosthesis stems such as the Link MP femoral reconstruction prosthesis allow rotational and axial control of the implant in the isthmus of the diaphysis. These implants can be used for revision of loosened femoral stems with extensive bone resorption of the proximal femur, enlargement of the medullary cavity, or extreme thinning of the cortical bone in the proximal region of the femur.

According to the manual for the implant (Link 2005), it is necessary to select a prosthesis stem size that will allow a form-fitting femoral distal anchorage for a minimum of 80 mm. This distance is believed to be required to achieve stable prosthesis anchorage. However, this length of anchorage is based on theoretical assumptions only (personal communication with the manufacturer). Moreover, there have been no in vivo studies showing that an implant-bone anchorage of 80 mm can be achieved on a routine clinical basis and that this fixation length is necessary for implant stability and satisfying clinical results.

We thus assessed the distal femoral stem-bone anchorage of the Link MP hip reconstruction prosthesis using computer-assisted tomography (CT). In addition, we studied the relationship between distal implant anchorage length and clinical outcome.

## Patients and methods

### Femoral hip reconstruction prosthesis

**Figure 1.**
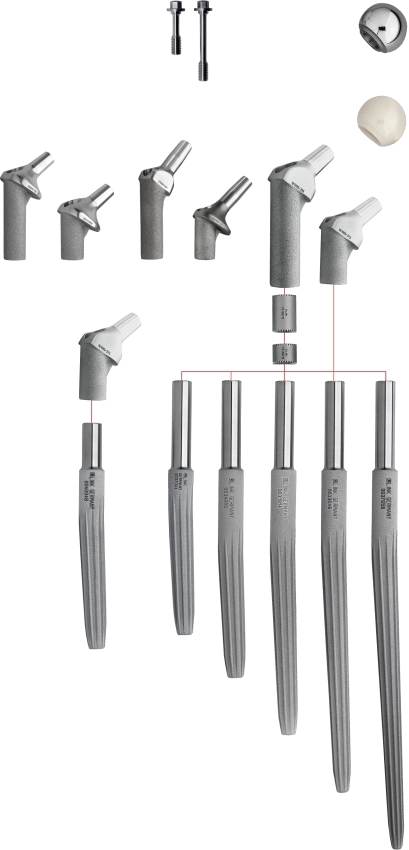
The Link MP hip revision arthroplasty showing options for different stem lengths, proximal bodies, and spacers to further adjust leg length.

The MP reconstruction implant consists of a modular cementless femoral hip stem, which is constructed of titanium (Ti6A14V) alloy with a microporous surface averaging 70 μm (Figure 1). The tapered stem has a fluted geometry with a 3° angular bow to accommodate the femoral curvature. The MP prosthesis is impacted into the femur until rigid stability to axial and torsional testing is achieved. Varying stem lengths and diameters allow independent fitting of the diaphysis. The modularity of the implant affords variability in neck geometry. Depending on the stem size, the MP prosthesis contains 8 or 10 longitudinal flutes (stem sizes 12–16 and 18–25, respectively) to support rotational stability and to reduce the stiffness of the implant (Link 2005).

### Patients and clinical assessment

We included 14 consecutive patients who had undergone hip revision surgery with the MP reconstruction stem and who were willing to undergo CT examination. We did not select patients according to clinical or radiographical outcome.

The median age of the patients at revision surgery was 78 years (interquartile range, IQR: 63–81) (Table). Regarding the Charnley classification (Roder et al. 2006), 3 patients were grouped as class A (unilateral total hip arthroplasty (THA), contralateral hip not diseased), 2 patients as B (unilateral THA, contralateral hip diseased) and 9 patients as BB (bilateral THA).

**Table 1.** Demographic data on patients with the Link MP reconstruction prosthesis

Patient no.	Sex	Age	Distance of stem-bone anchorag e (mm) **^a^**	Pain at rest (mm) **^b^**	Pain with movement (mm) **^b^**	Thigh pain (yes/no)	Distal stem migration (mm)	Proximal bone remodeling	Stem length (mm)	Stem diameter (mm)
1	F	60	75	5	4	no	0	A	210	16
2	M	81	40	0	0	no	0	A	250	18
3	F	82	15	3	8	no	3	B	210	16
4	M	80	10	3	4	no	0	B	250	18
5	F	79	55	3	5	no	0	A	210	16
6	F	79	50	0	0	no	0	B	210	18
7	F	62	50	3	5	no	0	A	210	14
8	F	84	10	0	30	yes	2	A	210	18
9	F	69	10	0	0	no	1	C	250	18
10	M	63	10	0	0	no	0	C	250	18
11	F	64	75	0	0	no	0	A	210	14
12	M	77	95	0	0	no	0	B	250	18
13	F	52	15	0	0	no	0	C	250	14
14	F	79	25	5	5	no	8	C	250	20

^a^ Assessed by computer-assisted tomography.

^b^ Visual analog scale (0–100 mm; 0 no pain).

A, increasing defects; B, constant defects; C, osseous restoration.

F, female; M, male;

All patients were operated by a posterolateral approach. At revision surgery, 1 Mueller, 1 Exeter, and 12 Charnley cemented hip prostheses with a median survival time of 12 years (IQR: 8–12) were extracted due to aseptic loosening. In 10/14 cases, acetabular revisions with the Mueller reinforcement ring (Zimmer Inc., Warsaw, IN) and a corresponding cemented polyethylene cup within the ring were performed.

At a median follow-up time of 12 months (IQR: 12–25), we performed spiral CT scans on the patients' operated femora to assess distal stem-bone anchorage. A clinical evaluation was performed both at 12 months (IQR: 12–25) and at 62 months (IQR: 61–73) follow-up.

Demographic data were recorded for all study subjects. Clinical evaluation included an assessment of thigh pain (yes/no), pain at rest, and movement in the hip joint (0–100 mm visual analog scale, where 0 = no pain). In addition, the Harris hip score (Harris 1969), the Merle d'Aubigné score (D'Aubigne and Postel 1954) and the Trendelenburg's sign were assessed. The postoperative regimen consisted of toe-touch weight bearing for 2 months and subsequent progressive weight bearing as tolerated.

The study design was approved by the local Ethics Committee of Stockholm North (DN 02-064).

### Radiographic analysis

**Figure 2.**
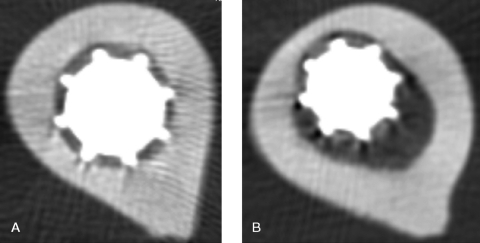
Computer-assisted tomography showing transverse planes of different levels of a patient's femur with a cementless Link MP reconstruction prosthesis. The stem contains 8 longitudinal distal flutes to provide rotational stability. More (A) and less (B) than 50% of the flutes have cortical bone contact.

CT scans were performed with a protocol using Picker PQ 5000 single-slice (Picker International, Cleveland, OH) or GE lightspeed 16-slice (GE Healthcare) CT machines according to availability. The single-slice protocol used 120 kV, 175 mA, pitch 2, and rotation of 1 second. The 16-slice protocol used 120 kV, pitch 1.375, a rotation time of 0.6 second and a noise index of 15, yielding approximately the same radiation dose for the patients and equivalent picture quality. Pictures were reconstructed with a 5-mm interval without overlap (Figure 2A and B). All CT scans were analyzed separately by 2 blinded radiologists. Intraclass correlation coefficient was used to measure inter-rater reliability.

In order to achieve predictable prosthesis fixation, it is best to implant the stem with a tight press-fit at the femoral isthmus. Poorly fitting stems are less likely to become fixed by bone ingrowth. The femoral stem-bone anchorage was defined as being adequate in a CT slice if 50% or more of the stem flutes had cortical bone contact. The length of anchorage was derived from the number of slices with adequate anchorage (Figure 2A and B). The theoretical basis for this assumption is that in this way, the opposite side of the femur is pressurized against the implant and stability is achieved through press-fit.

The radiographs taken immediately after the index operation were compared with those at follow-up examination in order to classify the restoration of the proximal part of the femur and the degree of subsidence. Bone remodeling of the proximal femur was classified subjectively as A (increasing defects), B (constant defects), or C (osseous restoration) (Bohm and Bischel 2001).

Distal migration of the femoral component of more than 5 mm was defined as subsidence (Sporer and Paprosky 2004). Subsidence landmarks were fixed points on the prosthesis, such as the lesser trochanter and cerclage wires if present. We accounted only for radiographic changes on the anterior-posterior view, because the variable quality of the lateral view, especially at the postoperative examination, made evaluation of this view uncertain. Radiographs were assessed digitally using Sectra PACS software IDS5 (Sectra-Imtec AB, Sweden). A coefficient—the ratio of the actual diameter and the measured diameter of the femoral head—was calculated for adjustment of magnification (Salemyr et al. 2008).

### Statistics

All data are given as median with interquartile range (IQR), which gives the numerical difference between the twenty-fifth and seventy-fifth percentiles. Spearman's rank correlation coefficient was used to evaluate the association between the Harris hip score, the Merle d'Aubigné score, pain scores, and the distance of distal femoral stem-bone anchorage. The Wilcoxon signed-ranks test was used to compare the patients' median distance of stem-bone anchorage with 80 mm. A p-value of ≤ 0.05 was considered statistically significant. All statistical analyses were performed using Stata 9 (StataCorp LP, College Station, TX).

## Results

### Clinical data

At the 12-month follow-up (IQR: 12–25), all patients were fully weight-bearing and only 1 patient complained about occasional mild thigh pain. The median for VAS pain was at rest, 0 (IQR: 0–3), and with movement, 2 (IQR: 0–5) (Table). For the Harris hip score (HHS), the patients had a median of 85 points (IQR: 77–94), which is considered a good result according to the original article (Harris 1969). For the Merle d'Aubigné score, the patients had a median of 10 points (IQR: 8–12), corresponding to a good outcome. The Trendelenburg's sign was positive in 4, negative in 4, and classified as being unsure in 6 patients.

At the 62-month follow-up (IQR: 61–79), the median for VAS pain at rest was 0 (IQR: 0–1) and with movement it was 0 (IQR: 0–14). The Harris hip score reached a median of 81 points (IQR: 62–94) and the Merle d'Aubigné score reached 9 points (IQR: 6–12). None of the stems had to be revised for any reason.

### Radiographic findings

CT delivered implant and femoral pictures of good quality, which made it easy to analyze the CT scans. The median length of distal femoral stem-bone anchorage was 33 mm (IQR: 10–60), which was significantly shorter than 80 mm (p = 0.002) (Table). Intraclass correlation coefficient between the measurements of the two blinded radiologists was 0.94, corresponding to an outstanding inter-rater reliability. There was 1 patient with stem subsidence (7%; 95% CI: 0–34). Concerning bone remodeling, 8 patients showed constant defects or osseous restoration of the proximal femur (Table).

At the 12-month follow-up, there was a correlation between the length of femoral stem-bone anchorage and the HHS (r = 0.56, p = 0.04). There was no significant correlation between stem-bone anchorage and the Merle d'Aubigné score, pain scores at rest or movement, distal stem migration, or bone remodeling (–0.5 < r < 0.4, p > 0.05).

At the 5-year follow-up, there was no statistically significant correlation between the stem-bone anchorage and any of the clinical or radiographic scores (–0.1 < r < 0.4, p > 0.05).

## Discussion

We found a discrepancy between clinical practice and the accepted guidelines concerning stem-bone anchorage of the cementless MP reconstruction prosthesis (Link 2005). The length of distal femoral fixation achieved in routine clinical practice was shorter than recommended by the manufacturer. We found it difficult to achieve an implant anchorage length of at least 80 mm on a standard femur with proximally compromised bone stock. However, on the basis of our results, we do not see the necessity for such a long stem-bone anchorage to achieve a good clinical and radiographical outcome.

All patients in our cohort were fully weight-bearing. Half of the patients did not experience any hip pain at rest or movement, and only 1 patient complained about mild thigh pain. Our cohort had HHS and Merle d'Aubigné score indicating good results. Apart from there being a moderate degree of correlation between HHS and anchorage length, there was no further association between clinical outcome, radiographic scores, and length of stem-bone anchorage.

A frequently reported complication of revision hip stems is subsidence (Engh et al. 1987, Krishnamurthy et al. 1997). To avoid migration, a solid fixation of the implant into the femoral isthmus of a minimum of 40 mm is recommended by some authors (Jones 2004, Krishnamurthy et al. 1997, Paprosky et al. 1999). In our study, we found a median anchorage length of 33 mm, which is in line with the findings mentioned above. Even with this short distance of fixation, we detected only 1 patient with stem subsidence at follow-up.

Several studies evaluating fluted tapered implants such as the modular MP stem (Bellomo et al. 2002, Kwong et al. 2003) or the monoblock Wagner SL revision stem (Grunig et al. 1997, Isacson et al. 2000, Bircher et al. 2001, Bohm and Bischel 2001) have shown clinical efficacy and reliable results in revision femoral surgery. The MP stem may even be used in periprosthetic femoral fractures giving good implant stability, good clinical results, and excellent fracture healing (Berry 2003). The modular stem must be strong enough to support high loads, possibly for long periods even in the absence of good proximal bone support (Postak and Greenwald 2001). The fact that the patients in this study functioned well in the short term and the fact that the cementless stems showed no radiographic signs of loosening suggest successful implant stability with biological stem fixation.

The purpose of this study was not to perform a clinical medium-term follow-up of patients with a cementless hip revision arthroplasty. However, we found it of interest to re-assess our cohort clinically after 5 years, and could show that their outcome had not deteriorated. At the time of the study, we had a rather conservative postoperative regime with 2 months of toe-touch weight bearing. Today, patients are immediately allowed to put weight, as tolerated, on the operated extremity.

This study had several possible shortcomings. The relatively low sample size reduced the actual statistical precision. We have no clinical long-term follow-up of our cohort, and the CT method is not validated. The idea that at least 50% stem-bone contact is needed for implant stability is based on the fact that in such a way, opposing sides are pressurized against each other. However, this is a theoretical assumption. So far, there have been no biomechanical or cadaver studies to support this.

Due to the lack of digital radiograph templates at the time of the study we did not choose the appropriate implant size preoperatively. However, the preoperative radiographs show the situation before the extraction of the primary prosthesis and cement. The intraoperative situation may be different from the preoperative radiographs, and so the planning may only give a very rough impression concerning prosthesis size. All patients in our cohort were operated by 2 experienced hip revision surgeons who decided upon implant size intraoperatively, based on proper fit and stability.

Despite these limitations, our results point in the same direction, i.e. that there were good clinical results and implant stability, and thus a short distal stem anchorage. We found the CT scans to be of good quality and free of disturbing metal artefacts, which made it easy to interpret the images. The CT measurement itself is reliable, which can be seen from the inter-rater agreement. Moreover, evaluation of cementless implants with CT has been shown to be a valid method in other studies (Jedenmalm et al. 2008, Olivecrona et al. 2008).
